# Development and comparative analysis of initiation ability in large-scale *Heuchera* propagation using tissue culture versus cuttings

**DOI:** 10.1038/s41598-023-42001-8

**Published:** 2023-09-07

**Authors:** Chan Xu, Hang Guo, Zhijing Wang, Yuan Chen

**Affiliations:** Institute of Vegetables and Flowers, Chongqing Academy of Agricultural Sciences, Chongqing, 400000 China

**Keywords:** Plant sciences, Plant reproduction

## Abstract

The *Heuchera* genus, a member of the *Saxifragaceae* family, encompasses a wide array of varieties and hybrids, serving both traditional medicinal and ornamental purposes. However, a significant knowledge gap persists in achieving efficient mass propagation of diverse *Heuchera* cultivars creating a substantial market void. To address this, our study focuses on expedited seedling regeneration by investigating leaf cutting and tissue culture techniques to offer novel insights to cultivators. Herein, we successfully rooted thirteen distinct cultivars from the *Heuchera* and *Heucherella* (*Heuchera* × *Tiarella*) genera through cutting. Moreover, in vitro culture experiments led to the successful induction of calli and shoots from petiole samples. Notably, variations in measured parameters were observed across cultivars in both cutting and tissue culture methodologies. When petiole explants were exposed to cytokinin 6-benzylaminopurine (BA) at concentrations of 0.5, 1.0, and 2.0 mg/L along with auxin α-naphthaleneacetic acid (NAA) at 0.5 mg/L, shoots were produced either directly or indirectly during the primary culture. Exposure to darkness and the application of 2,4-dichlorophenoxyacetic acid (2,4-D) did not promote shoot formation but were beneficial for callus stimulation. Interestingly, a negative correlation was observed between the ease of initiating cutting recovery and inducting tissue culture regeneration, suggesting that cultivars that easily regenerate through cutting might encounter difficulties during induction by tissue culture. In light of these findings, we devised a streamlined and effective protocol for rapid *Heuchera* propagation. This protocol involves micropropagation, directly acquiring adventitious shoots from primary cultures supplemented by cutting-based propagation methods.

## Introduction

The genus *Heuchera*, a member of the *Saxifragaceae* family encompasses a wide range of varieties and hybrids, with new additions every year. *Heuchera,* also known as Alum root or Coral bells, is a perennial herbaceous plant native to North America, capable of enduring temperatures as low as – 30 °C. While the *Heuchera* has historically been employed medicinally for various applications such as reducing tissues in nosebleeds, sore throats, ulcers, diabetes, and hemorrhoids^1^, its numerous hybrid cultivars are now extensively utilized across North America, Europe, Africa, and Asia for their ornamental properties^2^. *Heuchera* displays ornamental value with its incredible variety of foliage colors and shapes. The foliage and venation come in colors like purple, rose, lime green, and gold, and the foliage shapes can be rounded, lobed, and hairy. These plants are grown in woodlands, rock gardens, containers, borders, and ground covers. Heuchera cultivars are in high demand worldwide as ornamental foliage plants, but they are short-lived perennials that may die out in just a few years if not divided regularly. Therefore, ensuring the preservation of *Heuchera* genetic resources and market supply is a major challenge for breeders and plant geneticists.

While conventional methods of propagation involve splitting and sowing, *Heuchera* can theoretically be propagated through sowing, cutting, dividing, and tissue culture. Nevertheless, certain propagation techniques come with inherent limitations. Seed propagation, for instance, is a cost-effective approach but is hindered by low seed production and substantial genetic variation post-sowing. Similarly, division propagation’s pace is insufficient for large-scale reproduction, yielding 10–15 plantlets annually from a mother plant^3^. Furthermore, numerous varieties exhibit limited ramet production potential, and division can detrimentally impact root systems, diminishing survival rates. Cutting and tissue culture provide viable alternatives to retain distinctive phenotypic attributes and generate numerous offspring. Among them, tissue culture culture provides a basic tool for many applied technologies. However, only handful of *Heuchera* varieties have been previously studied in vitro, such as *Red Spangles*^3^, *Caramel*^4^, *Silver Scrolls*^5^, etc.

Despite the abundance of *Heuchera* cultivars and their widespread popularity, information on effective propagation methods remains limited. The present study sought to help producers obtain seedlings in the shortest regeneration period, thereby minimizing production costs. We examined clonal propagation methods like cutting and in vitro culture, which depend on cell totipotency. Given this shared dependency, we questioned whether cultivars that easily initiate cutting propagation also readily initiate in vitro culture. However, the correlation between these methods has not been empirically studied before. Thus, we investigated cutting propagation and histological differentiation on 13 diverse *Heuchera* and *Heucherella* (*Heuchera* × *Tiarella*) cultivars with varying foliage colors and venation patterns.

## Materials and methods

The study complied with local and national regulations.

### Cultivars and cutting propagating by leaf cuttings

This research was conducted at the Plant Tissue Culture Factory of the Agricultural Science and Technology Innovation Center (Latitude: 29.8045° N, Longitude: 106.2932° E), situated in Bishan National Agricultural Science Park, Chongqing, China. The mother plants of *Heuchera* and *Heucherella* under investigation were sourced from TianMu Agriculture Co., Ltd. (Chongqing, China) and were bred by Terra Nova Nurseries, INC. (Oregon, USA); their traits are detailed in Table 1. Leaf cuttings, each containing intact petioles, were obtained from one-year-old mother plants cultivated in a greenhouse adjacent to the tissue culture laboratory. All leaf cuttings were placed in 50-cell plug trays (measuring 4.8 cm in top diameter, 1.8 cm in bottom diameter, and 8.5 cm in height), filled with a mixture of peat soil and perlite (3:1), and were grown in the greenhouse. During the study period, a fertilizer (Stanley Fertilizer Co., LTD, Shandong, China) was sprayed onto the leaves twice weekly.

**Table 1:** List of the *Heuchera* cultivars used in this study.

Cultivars	Genus	Height × Spread	Foliage description
Blonde	*Heuchera*	12 cm × 20 cm	Caramel-colored leaves flushed copper on the upper surface and with lobed edges
Electra	*Heuchera*	25 cm × 30 cm	Large lobed leaves, bright yellow with blood-red veins, chartreuse in summer, tan in winter
Electric Lime	*Heuchera*	45 cm × 40 cm	Large lobed lime-tinted leaves with red veins in cooler temperatures
Forever Red	*Heuchera*	20 cm × 35 cm	Cut softly ruffled leaves with red veins
Lime Marmalade	*Heuchera*	25 cm × 60 cm	Heavily ruffled and frilled lime green leaves
Lime Ricky	*Heuchera*	20 cm × 45 cm	Lobed, rounded leaves with ruffled edges, lime-green to chartreuse color
Paris	*Heuchera*	45 cm × 40 cm	Lobed leaves, mint-green, overlaid with silver markings
Plum pudding	*Heuchera*	20 cm × 40 cm	Glossy, ruffled plum purple leaves with deep purple undersides and darker purple veins
Rio	*Heuchera*	25 cm × 50 cm	Peach-amber Leaves with pink veins
Shanghai	*Heuchera*	45 cm × 40 cm	Silvery-grey, Reddish-purple in All seasons;
Sweet Tart	*Heuchera*	20 cm × 20 cm	Very tangy, lime-colored foliage in a tight mound
Sweet Tea	*Heucherella*	50 cm × 60 cm	Maple-like, palmately lobed apricot orange leaves with contrasting burgundy veins
Tapestry	*Heucherella*	20 cm × 40 cm	Dark-centered blee-green leaves with purple veins

### Explant materials and sterilization

Petiols explants were taken from healthy mother plants of the cultivars listed in Table 1. The selected petiole sections were thoroughly washed under running tap water to remove dirt and dust; then surface moisture was removed using filter paper. The cleaned petioles were surface-sterilized by soaking in 75% ethanol for 30–60 s, followed by 2% sodium hypochlorite for 10–20 min, and finally rinsed three times with sterile water. Both ends of the sterilized petioles were then removed and discarded. The remaining petiole sections were cut into small segments (1–2 cm) for explant inoculation. Three to five of these segments were inoculated into each culture bottle. The experiment was repeated three times, with over 80 explants of each variety inoculated in each repeat.

### Primary culture medium and conditions

The culture media used were based on MS (Murashige and Skoog, 1962)^6^, supplemented with 3% sucrose and 6.5% agar, and adjusted to a pH of 6.0 ± 0.2. Various combinations of plant growth regulators (PGRs) were incorporated, including 6-benzylaminopurine (BA) as a cytokinin and α-naphthaleneacetic acid (NAA) as an auxin. The specific PGR combinations included concentrations of BA (0.5, 1.0, 2.0, and 4.0 mg/L) along with 0.5 mg/L NAA. The prepared media were autoclaved at 121 °C for 20 min. Cultures were maintained in a culture room with conditions set at 25 ± 1 °C and a 16 h light/8 h dark photoperiod. A dark pre-culturing step was introduced for cultivars exhibiting callus induction rates below 80% with the aforementioned PGR combinations after 30 days of primary culture. Specifically, *Blonde*, *Electra*, *Electronic Lime*, *Forever Red*, *Lime Ricky*, *Sweet Tart*, and *Sweet Tea* cultivars underwent dark culture. Dark culture was implemented for 0, 5, 10, and 15 days to identify the optimal period for improving callus induction rates. The cultivars were transferred back to the previously mentioned culture conditions after dark treatment. PGRs at 0.5 mg/L BA plus 0.5 mg/L NAA were utilized for dark culture.

Furthermore, the aforementioned screened cultivars were subjected to in another primary culture experiment. Based on the outcomes of the prior primary culture, attempts were made to enhance induction rates by adjusting the ratio of NAA or by substituting NAA with 2,4-D (2,4-dichlorophenoxyacetic acid). The PGR combinations involved 0.5 mg/L BA + 0.5 mg/L NAA, 0.5 mg/L BA + 1.0 mg/L NAA, 0.5 mg/L BA + 0.5 mg/L 2,4-D, and 0.5 mg/L BA + 1.0 mg/L 2,4-D.

### Statistical analysis

For the leaf cutting experiments, we recorded the rooting rate, root length, and number of roots after 50 days of growth. For the tissue culture experiments, we first observed the time of callus initiation, then recorded the callus induction rate on day 30 and shoot regeneration frequency on day 60 after inoculation. All data were analyzed and graphed using R software (version 4.3.1). ANOVA was performed using the ‘stats’ package with a fitted ANOVA model. Mean comparisons were conducted using Duncan's new multivariate range test at a 5% significance level via the ‘agricolae’ package. Correlation analysis between individual cutting and tissue culture parameters was performed using Pearson’s correlation in the ‘PerformanceAnalytics’ package.

## Results

### Initiation of cutting and tissue culture

**Figure 1:**
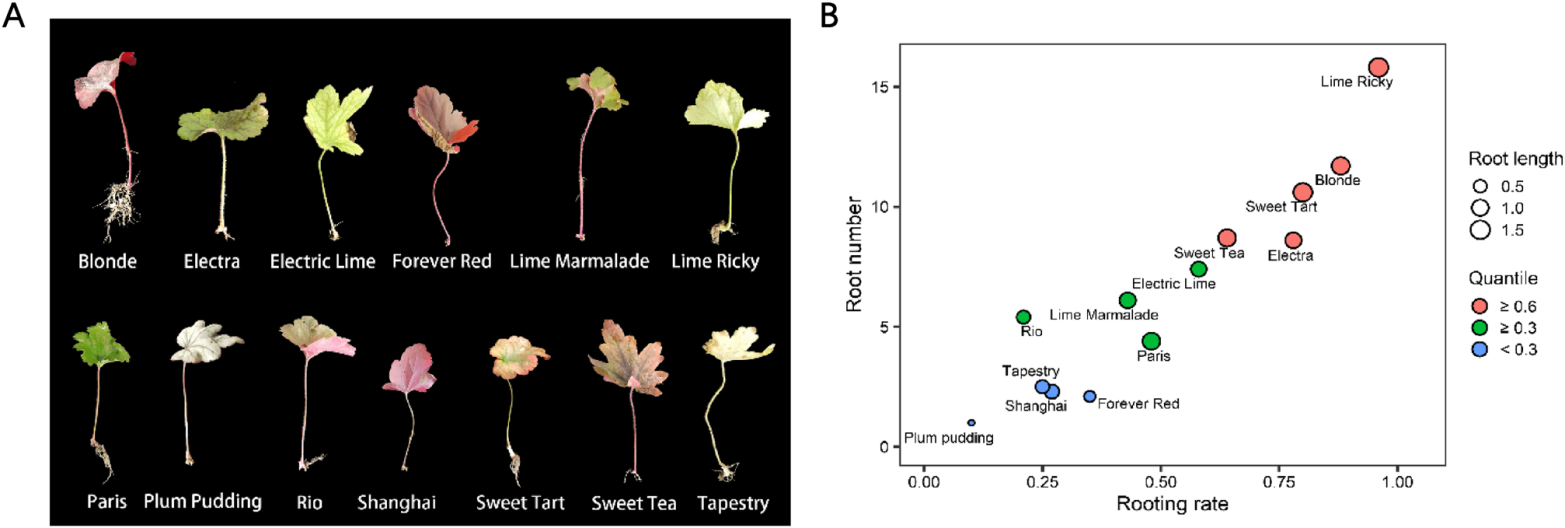
Rooting responses of different cultivars to leaf cutting after 50 days of growth. (**A**) Rooting status of each cultivar after cutting. (**B**) Dot plot illustrating the index for each cultivar and its leaf cutting regeneration potential. The distribution of points from the bottom left to the top right indicates increasing regeneration potential. The dot color represents the quantile calculated based on root number, length, and rooting rate.

**Figure 2:**
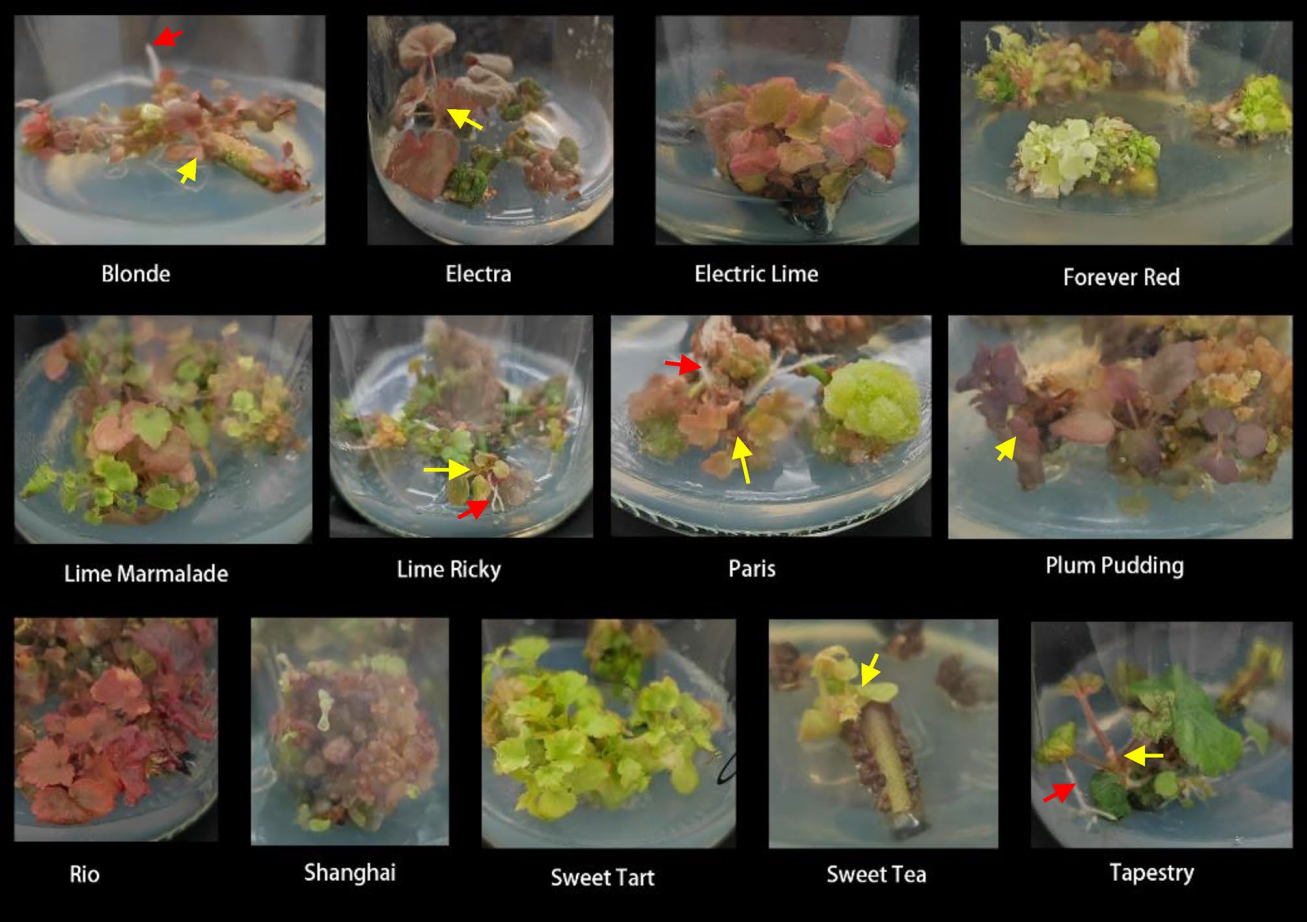
Responses of different cultivars to the In vitro culture. Red arrows: roots; yellow arrows: directly shoots.

Numerous cultivars from the *Heuchera* genus including *Blonde*, *Electra*, *Electric Lime*, *Forever Red*, *Lime Marmalade*, *Lime Ricky*, *Paris*, *Plum Pudding*, *Rio*, *Shanghai*, *Sweet Tart*, along with cultivars form the *Heucherella* genus including *Sweet Tea* and *Tapestry*, were effectively initiated both through cutting propagation (Fig. 1) and in vitro culture (Fig. 2).

### Rooting of leaf cuttings

Although all 13 cultivars successfully initiated rooting through cutting (Fig. 1), notable variations in rooting responses were observed among the cultivars. Following the cutting procedure, *Lime Ricky*, *Blonde*, *Sweet Tart*, *Electra*, and *Sweet Tea* exhibited rooting rates of 96%, 88%, 80%, 78%, and 64%, respectively, while the remaining cultivars exhibited rates below 60% (Fig. 1B). *Lime Ricky*, *Blonde*, and *Sweet Tart* displayed more than ten roots, while the remaining cultivars had fewer roots (Fig. 1B). Among the cultivars, five displayed roots longer than 1.0 cm, while the rest exhibited shorter roots (Fig. 1B). To assess relative regeneration potential, the three indices were normalized and averaged to obtain scores for each cultivar. Based on the quantile of these scores, the sequence of cutting regeneration potential was: *Lime Ricky*, *Blonde*, *Sweet Tart* > *Electra*, *Sweet Tea* > *Electric Lime*, *Lime Marmalade*, *Paris* > *Rio*, *Tapestry* > *Shanghai*, *Forever Red*, *Plum Pudding* (Fig. 1B).

### Responses of cultivars during in vitro culture

**Figure 3:**
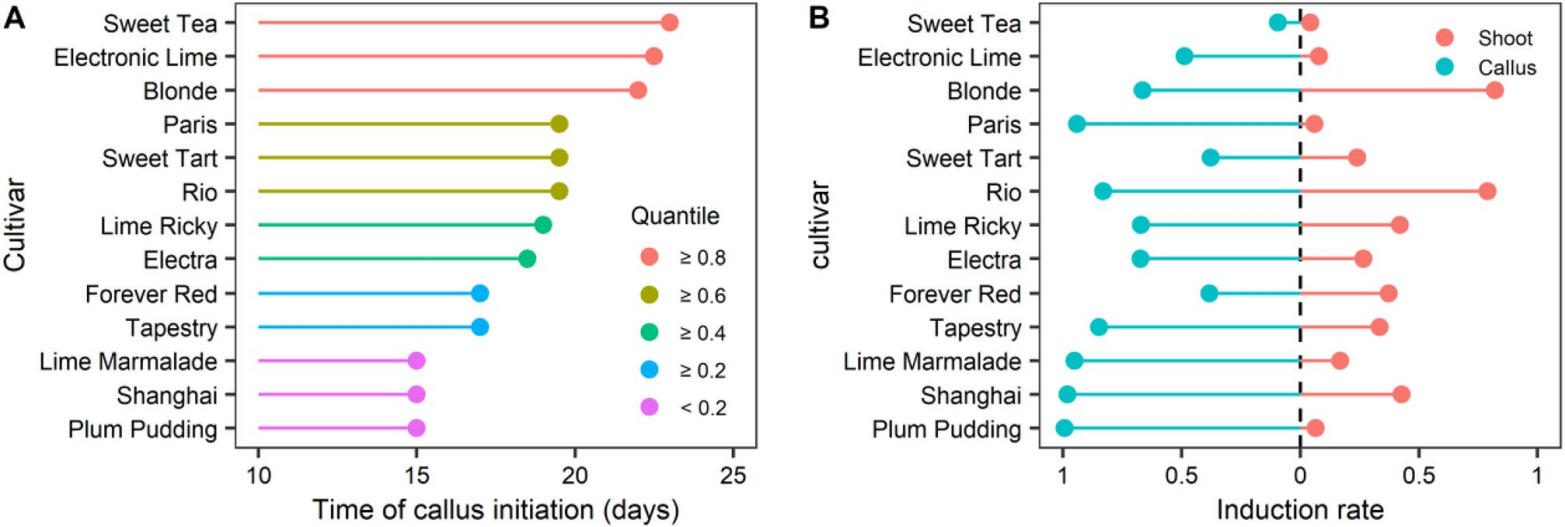
Comparison of time of callus initiation and callus induction rate and shoots induction rate in 0.5 mg/L BA + 0.5 mg/L NAA treatment. (**A**) Days of callus initation. (**B**) Induction rates of callus and shoots.

During primary *in vitro* culture, both calli and shoots were observed in each cultivar, with shoots emerging from the incised callus (Fig. 2). Diverse cultivars displayed variable callus induction and shoot formation when exposed to an MS medium containing BA and NAA. Callus initiation spans ranged from 15 to 23 days for the different cultivars, with three cultivars showcasing the swiftest callus initiation at 15 days (Fig. 3A). Notably, Tables 2 and 3 indicate significant variations in callus induction rates among six cultivars and shoot formation across three cultivars, based on differing BA concentrations. After 30 days of incubation, six cultivars achieved callus induction rates exceeding 80%, amounting to an efficiency of 46% (Table 2 and Fig. 3B). By the 60-day mark, *Rio* and *Blonde* cultivars exhibited significant shoot formation, reaching 83% (Table 3 and Fig. 3B).

**Table 2:** Effect of BA and NAA on callus formation in *Heuchera *in vitro.

Treatment		Callus formation frequency (%)												
BA (mg/L)	NAA (mg/L)	Blonde	Electra	Electronic Lime	Forever Red	Lime Marmalade	Lime Ricky	Paris	Plum Pudding	Rio	Shanghai	Sweet Tart	Sweet Tea	Tapestry
0.5	0.5	66.3 a	67.3 a	48.7 a	38.3 a	95.0 a	67.0 a	91.6 ab	99.3 a	83.0 a	98.0 a	37.7 a	9.3 a	84.7 a
1.0	0.5	74.7 a	67.0 a	21.5 b	51.7 a	92.0 a	55.0 a	93.7 a	98.0 ab	78.7 a	83.7 a	24.7 ab	9.0 a	72.7 ab
2.0	0.5	35.7 b	53.7 a	34.0 ab	53.3 a	95.3 a	44.7 a	80.7 c	97.0 ab	84.7 a	86.0 a	17.0 ab	8.7 a	58.7 bc
4.0	0.5	4.0 c	16.0 b	25.3 ab	35.0 a	82.3 c	48.7 a	82.0 bc	88.7 b	45.3 b	78.3 a	10.3 b	3.2 a	45.0 c
*F* value		68.2	14.6	2.6	0.7	18.7	0.9	3.9	3.0	6.3	1.8	2.4	2.5	7.2
Sig.		***	***	ns	ns	***	ns	*	ns	*	ns	ns	ns	*

BA, 6-benzylaminopurine; NAA, α-naphthaleneacetic acid.

Values in the same column annotated with different letters are significantly different (p < 0.05).

***, **, *, and ns indicate p value < 0.001, < 0.01, < 0.05, and > 0.05, respectively.

**Table 3:** Effect of BA and NAA on shoot formation in *Heuchera *in vitro.

Treatment		Shoot regeneration frequency (%)												
BA (mg/L)	NAA (mg/L)	Blonde	Electra	Electronic Lime	Forever Red	Lime Marmalade	Lime Ricky	Paris	Plum Pudding	Rio	Shanghai	Sweet Tart	Sweet Tea	Tapestry
0.5	0.5	78.7 a	19.0 a	5.3 a	28.7 a	21.7 a	29.3 a	9.6 a	6.7 a	61.3 a	58.0 a	27.3 a	5.0 a	22.0 a
1.0	0.5	83.0 a	8.0 a	0.7 b	28.0 a	3.7 b	21.7 a	0.5 c	2.7 a	56.3 a	41.5 a	17.0 ab	2.7 a	32.0 a
2.0	0.5	70.0 a	21.0 a	8.3 a	38.7 a	11.7 ab	36.0 a	2.0 b	6.5 a	83.3 a	38.3 a	8.3 b	2.7 a	32.3 a
4.0	0.5	13.0 b	21.3 a	0.5 b	15.0 a	0.3 b	36.3 a	0.5 c	2.7 a	61.3 a	30.7 a	2.7 b	2.7 a	35.0 a
*F* value		17.7	0.8	7.8	1.3	5.5	0.5	78.7	0.6	1.1	0.9	4.3	1	0.8
Sig.		***	ns	**i	ns	ns	ns	***	ns	ns	ns	ns	ns	ns

BA, 6-benzylaminopurine; NAA, α-naphthaleneacetic acid.

Values in the same column annotated with different letters are significantly different (p < 0.05).

***, **, *, and ns indicate p value < 0.001, < 0.01, < 0.05, and > 0.05, respectively.

**Figure 4:**
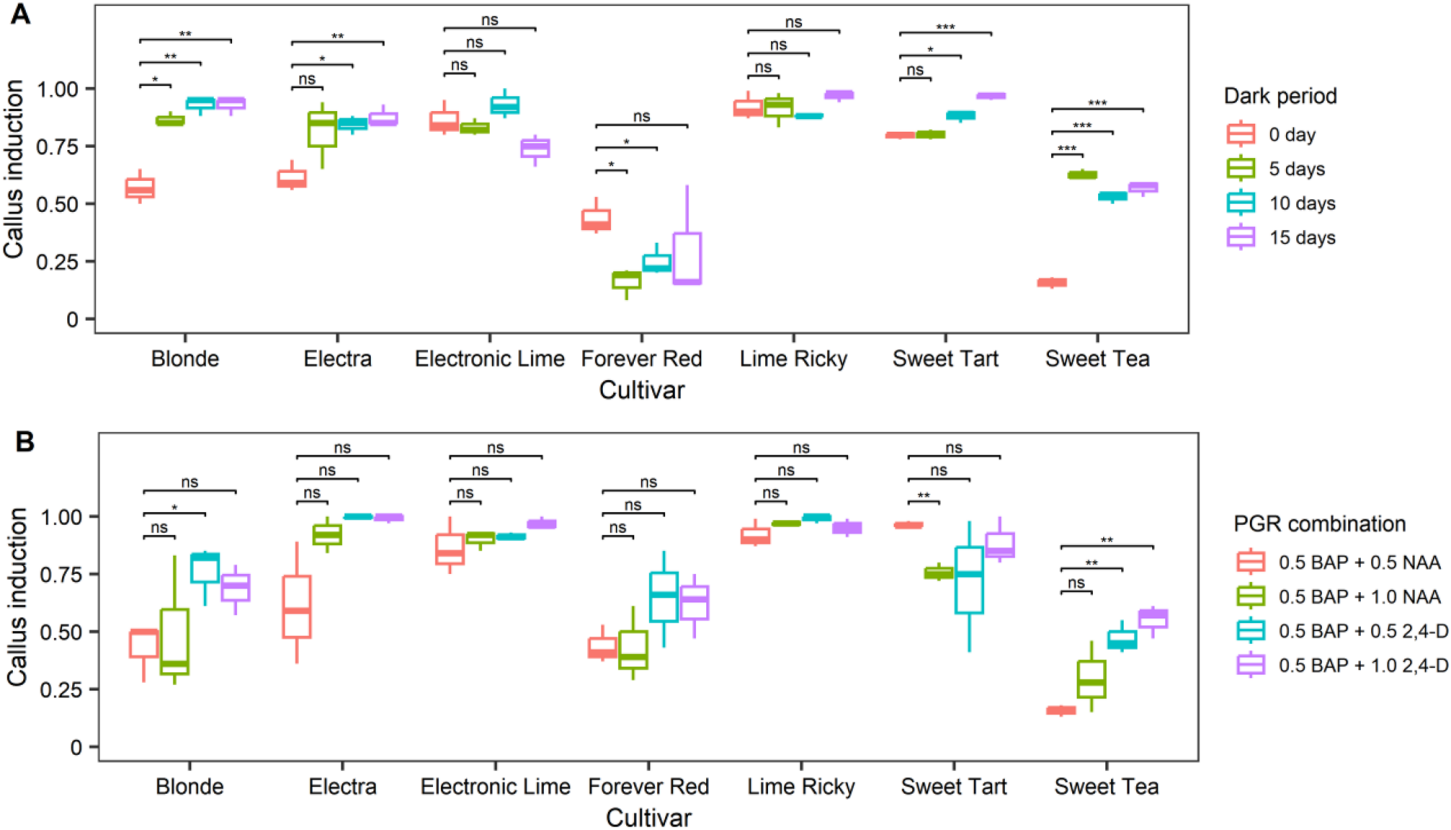
Effects of dark culture and modified PGR on callus induction. (**A**) Dark treatment with PGR combination 0.5 mg/L BA + 0.5 mg/L NAA. Explants without dark treatment were used as controls (0 day). Dark-treated explants with different durations (5 days, 10 days, 15 days) were analyzed for differences from controls and marked for significance. (**B**) Modified PGR combination. The 0.5 mg/L BA + 0.5 mg/L NAA combination was used as control, and the differences between other treatments and control were analyzed and marked as significant. ***, **, *, and ns indicate p value < 0.001, < 0.01, < 0.05, and > 0.05, respectively.

Seven cultivars with callus induction rates below 80% underwent dark treatment before a subsequent incubation attempt, including *Blonde*, *Electra*, *Electronic Lime*, *Forever Red*, *Lime Ricky*, *Sweet Tart*, and *Sweet Tea*. Callus induction rates for *Blonde* and *Sweet Tea* significantly increased after 5, 10, or 15 days of dark treatment (Fig. 4A). *Electra* and *Sweet Tart* displayed noteworthy improvements after 10 and 15 days of dark treatment, while *Electronic Lime* and *Lime Ricky* exhibited no significant changes (Fig. 4A). Notably, *Forever Red* experienced a decrease in callus induction rate (Fig. 4A). Moreover, these seven cultivars were subjected to a revised experiment involving diverse PGR combinations (with 0.5 mg/L BA + 0.5 mg/L NAA serving as the control). Compared to the control, treatment with 0.5 mg/L BA + 1.0 mg/L NAA did not substantially enhance induction rates across all cultivars (Fig. 4B). In the case of *Blonde* and *Sweet Tea*, induction rates significantly increased with the application of 0.5 mg/L BA + 0.5 mg/L 2,4-D, while other cultivars displayed negligible changes (Fig. 4B). Neither the dark pre-treatments nor the revised PGR treatments yielded improvements in shoot induction among the seven cultivars (Supplementary Fig. 1).

### Correlation analysis of cutting and tissue culture initiation

**Figure 5:**
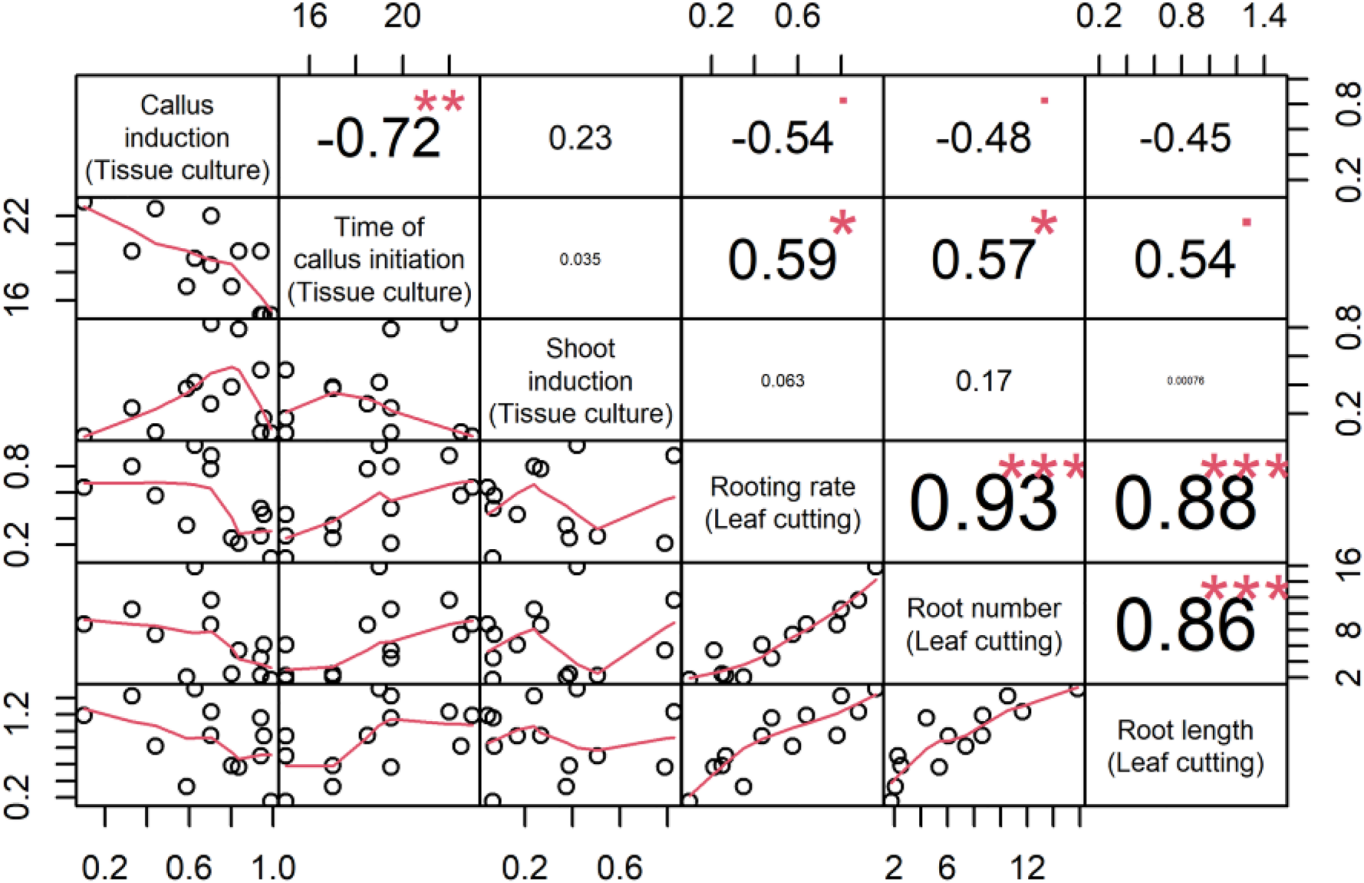
Correlation of indexes of cutting and in vitro culture. Statistical significance is indicated by *** p < 0.001; ** p < 0.01, and * p < 0.5 while correlation is indicated by Pearson’s correlation coefficient r.

Pearson correlation analysis was conducted between various measures and is presented in Fig. 5. There was a strong negative correlation (r = − 0.71) between callus induction rate and times of callus initiation. However, very weak or no correlations were found between shoot formation and callus induction or time of callus initiation. As expected, there were significant positive correlations among the cutting indexes (r ≥ 0.86).

The correlation between callus induction and cutting indexes is interesting because cutting indexes were negatively correlated with callus induction, positively correlated with the time of callus initiation, and only weakly or not correlated with shoot formation.

## Discussion

Although studies have reported successful shoot regeneration from shoots, leaves, and petioles of Heuchera cultured in vitro^3,5^, petioles have advantages as explants over other plant parts. It has been established that using whole shoots is detrimental to mother plants, and petioles regenerate more shoots than leaves^5^. Additionally, leaves as explants are more susceptible to contamination due to their larger surface area, making disinfection more challenging. Furthermore, direct and indirect shoot organogenesis from petiole explants has been documented in various plants^7,8^. Multiple *Heuchera* cultivars in this study successfully regenerated shoots from petiole culture, confirming petioles as excellent explants for *Heuchera *in vitro culture.

There is an increasing consensus suggesting that high cytokinin-to-auxin ratio promotes shoot regeneration^9–11^, while a low ratio stimulates roots^12^, and specific ratios induce callus formation^13,14^. Previous *Heuchera* studies used BA and NAA combinations^3,5^, with BA acting as cytokinin and NAA acting as auxin. Since we foucsed on shoot formation, BA/NAA ratios favoring cytokinin were used. Treated cultivars showed varying shoot regeneration, though callus formation rates exceeded shoot rates in most cases. Some studies have reported that 2,4-D with cytokinin improves regeneration^15^, though 2,4-D suppressed shoot formation in the present study, increasing callus in some cultivars, which is in agreement with the suggestion that shoots form at undetectable 2,4-D levels^16^, indicating a minimal 2,4-D threshold for *Heuchera* shoot regeneration. Hence, we employed 2,4-D as an alternative PGR for certain cultivars. Surprisingly, BA plus 2,4-D hindered shoot regeneration across all treated cultivars while increasing callus formation frequencies in some cultivars. These results align with Sandal's suggestion from 2005, speculating that different species' tissue culture morphogenesis stages may require distinct intracellular 2,4-D concentration thresholds^17^. It is highly conceivable that *Heuchera* shoot formation might require a low or even zero threshold for intracellular 2,4-D concentration. Considering these outcomes, we excluded this PGR combination for shoot regeneration.

While dark incubation has been shown to promote shoot regeneration in certain plants^18–21^, no in vitro studies on *Heuchera* explored the effects of dark treatment. Our study indicated that dark treatment favored callus formation but did not stimulate shoot regeneration in certain *Heuchera* cultivars. Dark culture has been linked to increased endogenous auxin accumulation^22^, potentially driving heightened callus formation in specific cultivars. Although enhanced endogenous auxin can reportedly promote shoot formation to some extent^23^, our findings contradict this prior assertion. This discrepancy may be attributed to the *Heuchera* genotype or the auxin overproduction induced by dark treatment, ultimately inhibiting shoot regeneration.

Clonal propagation through cutting or in vitro culture leverages the remarkable regenerative potential of plant cells and their capacity for cell division. Callus is commonly perceived as a mass of undifferentiated parenchyma cells^24,25^ that can form at the incision of cuttings and in vitro explants. It is now understood that plants recover via organogenesis, leading to the development of shoots or roots from callus^26^. Histological analysis of explants revealed that calli and organs originate from the incision point^27^. This observation led us to speculate about the relationship between cutting and in vitro propagation. Rooting results highlighted distinct recovery potentials among different cultivars, facilitating the ranking of the 13 cultivars. In tissue culture experiments, these cultivars produced both calli and shoots from petioles. The correlations between cutting and in vitro culture results yielded intriguing insights. Specifically, rooting from cuttings exhibited a negative correlation with callus induction in tissue culture, with no correlation evident regarding shoot formation—contrary to expectations. This divergence could stem from distinct endogenous mechanisms activated by varying cultural conditions. Our findings suggest that cultivars with lower difficulty in cutting propagation face greater challenges for tissue culture.

**Figure 6:**
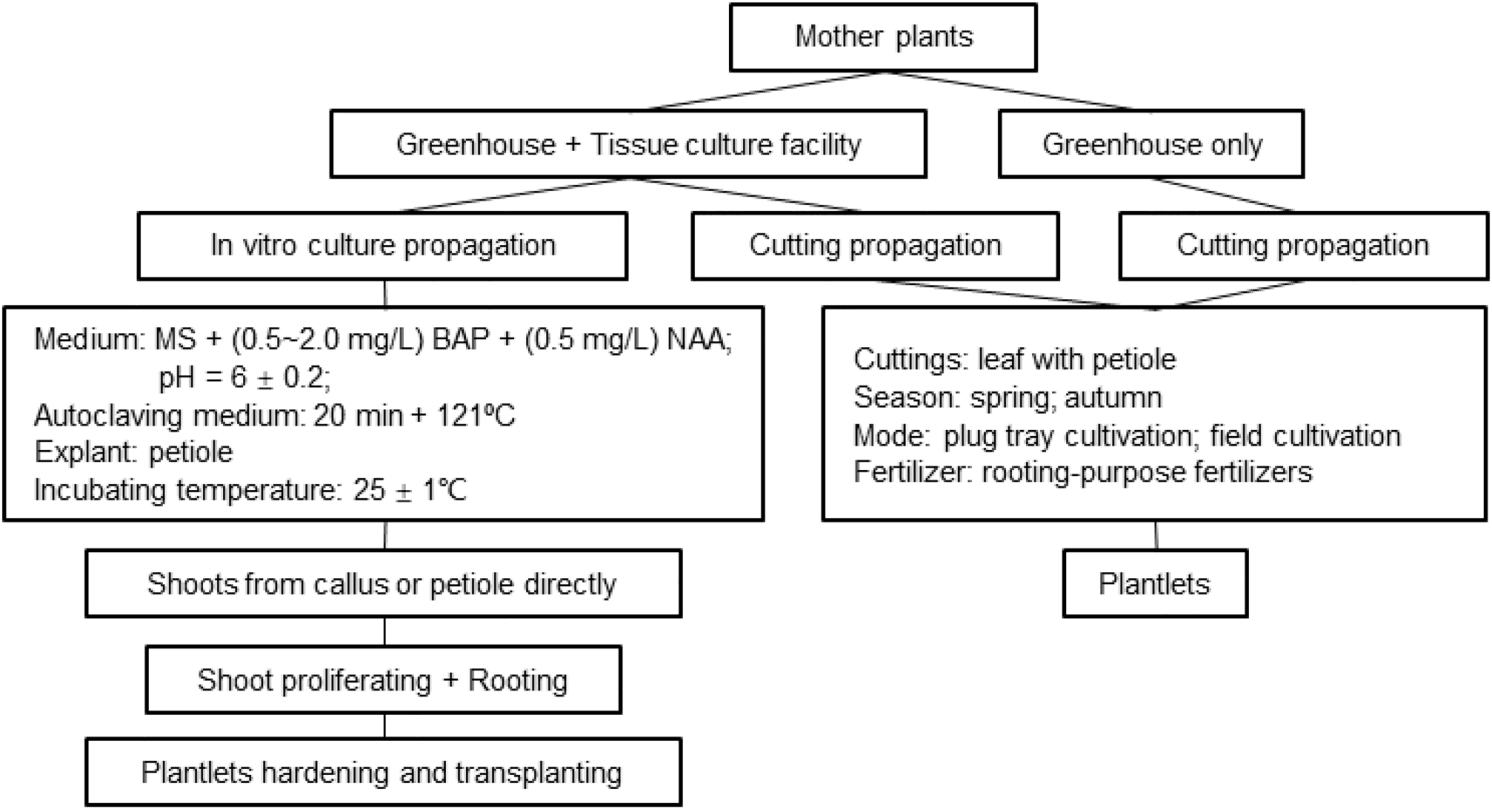
Technical route of rapid massive production.

Efficient *Heuchera* seedling production through cutting and in vitro culture methods is crucial for large-scale propagation. Based on our findings, we propose a potential protocol for Heuchera propagation, as outlined in Fig. 6. Several key points are worth noting: petioles are recommended as explants; the BA concentration should not exceed 4.0 mg/L; and 2,4-D should be avoided. Regardless of whether primary shoots are directly formed or developed from callus, both approaches yield shoots. The next step involves shoot proliferation to expand production capacity when primary shoots are obtained, regardless of the number.

## Conclusion

In the present study, plantlets were regenerated from petiole in vitro culture using either a two-step direct procedure—direct shoot formation and rooting—or a three-step indirect procedure involving callus induction, shoot development, and rooting. Petioles are recommended as explants, to be inoculated into MS medium with 0.5–2.0 mg/L BA and 0.5 mg/L NAA. We suggest utilizing comparatively lower BA concentrations due to the detrimental effect of 4.0 mg/L on induction. Moreover, 2,4-D should be excluded, given its inhibitory impact on shoot formation. Finally, we provide hitherto undocumented evidence of a negative correlation between cutting and tissue culture potential in *Heuchera*.

### Supplementary Information

Supplementary Figure 1.

## Data Availability

The datasets used and/or analyzed during the current study are available from the corresponding author upon reasonable request.
