# Supplementary material for: Development and comparative analysis of initiation ability in large-scale Heuchera propagation using tissue culture versus cuttings
Source: Sci Rep. 2023 Sep 7;13:14785. doi: 10.1038/s41598-023-42001-8 (PMC10484989; doi:10.1038/s41598-023-42001-8)
Supplement: Supplementary file 1 — Supplementary Figure 1. [file 41598_2023_42001_MOESM1_ESM.docx]

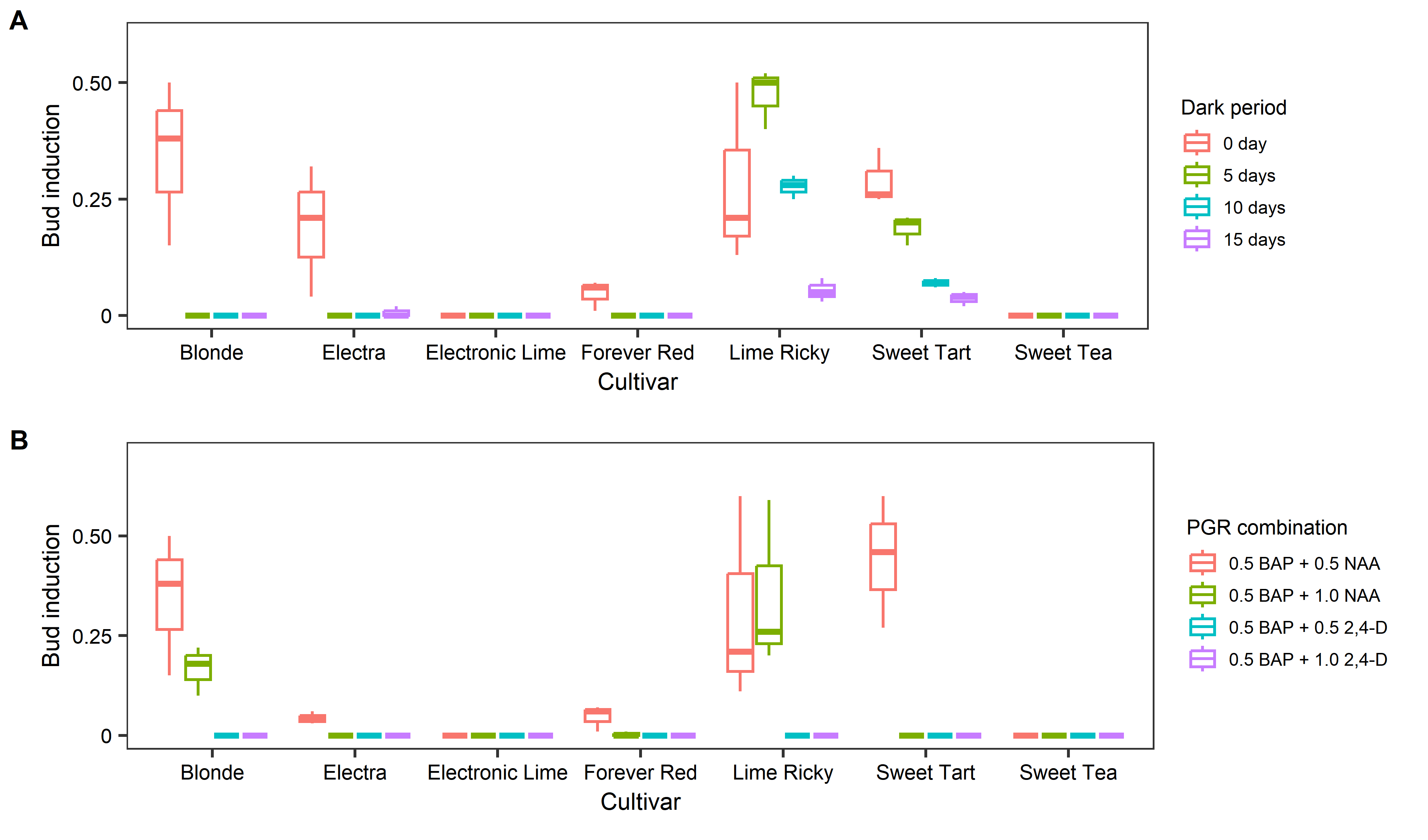


Supplementary Fig. 1 Effects of dark culture and modified PGR on shoot formation. (A) Dark treatment with PGR combination 0.5 mg/L BA + 0.5 mg/L NAA. Explants without dark treatment used as controls (0 day). Dark-treated explants with different durations (5 days, 10 days, 15 days) were analyzed for differences from controls and marked for significance. (B) Modified PGR combination. The 0.5 mg/L BA + 0.5 mg/L NAA combination was used as control.
